# Culture moderates changes in linguistic self-presentation and detail provision when deceiving others

**DOI:** 10.1098/rsos.170128

**Published:** 2017-06-07

**Authors:** Paul J. Taylor, Samuel Larner, Stacey M. Conchie, Tarek Menacere

**Affiliations:** 1 Department of Psychology, Lancaster University, Lancaster, UK; 2 Department of Psychology of Conflict, Risk and Safety, University of Twente, Enschede, The Netherlands; 3 Department of Languages, Information and Communications, Aston University, Birmingham, UK

**Keywords:** cross-cultural, language, deception, self-construal, memory

## Abstract

Change in our language when deceiving is attributable to differences in the affective and cognitive experience of lying compared to truth telling, yet these experiences are also subject to substantial individual differences. On the basis of previous evidence of cultural differences in self-construal and remembering, we predicted and found evidence for cultural differences in the extent to which truths and lies contained self (versus other) references and perceptual (versus social) details. Participants (*N* = 320) of Black African, South Asian, White European and White British ethnicity completed a catch-the-liar task in which they provided genuine and fabricated statements about either their past experiences or an opinion and counter-opinion. Across the four groups we observed a trend for using more/fewer first-person pronouns and fewer/more third-person pronouns when lying, and a trend for including more/fewer perceptual details and fewer/more social details when lying. Contrary to predicted cultural differences in emotion expression, all participants showed more positive affect and less negative affect when lying. Our findings show that liars deceive in ways that are congruent with their cultural values and norms, and that this may result in opposing changes in behaviour.

## 1. Introduction

People's use of language changes in subtle but distinguishable ways when they lie. Among other things, the statements of liars tend to include less contextual information, more negations and fewer first-person pronouns than the statements of truth-tellers [1–3]. However, while the literature on linguistic indicators of deception is extensive, almost all of what we know comes from research on Western subject populations. There is an implicit assumption that the processes that underlie the ‘leakage’ of cues to deception in these populations will apply universally across all cultures. But this assumption sits awkwardly with evidence of the profound role that culture can play in shaping interpersonal behaviour [4–7]. Culture affects the cognitive and affective factors that are theorized to cause differences in truth-teller and liars' behaviour. Evidence of culture moderating the traditionally observed differences will therefore provide independent support for these factors as being responsible for why behaviour changes during deception.

In this article, we make an initial contribution to our understanding of when and if culture affects deceptive behaviour by examining cultural differences in linguistic cues. Our focus on linguistic cues is relevant to several techniques that use features of the content of a statement to judge its likely veracity. These techniques include criteria-based content analysis (CBCA) [8] and scientific content analysis (SCAN) [1], which require coders to judge the characteristics of a statement based on the language used, and computer-based methods, which use text analysis to automatically identify and examine patterns in language use [9,10]. Many of these techniques are reported to be in use by law enforcement agencies worldwide (e.g. SCAN [1]), while others are being trialled for implementation [11,12]. Given this context, there is an urgent need to examine the cultural generalizability of using linguistic cues to infer a statement's veracity.

Our specific focus here is exploring the impact of individualism–collectivism on the occurrence of language-based indicators of deception. Understood as one of the primary dimensions by which cultures and their members can be differentiated, individualism–collectivism refers to the extent to which the characteristic values and norms of a society emphasize individuality and autonomy, or interdependence and connectedness [13,14]. At the individual level, these contrasting cultural syndromes have been shown to affect self-construal, emotion and remembering in ways that imply that language use during deception will not be culturally uniform. Indeed, as we argue below, the contrasting emphases lead us to hypothesize opposite changes in the occurrence of key indicators of deception, a prediction that is borne out in a comparison of genuine and fabricated statements from four ethnic groups.

### 1.1. Culture and linguistic indicators of deception

Although there are exceptions [15], one widely observed change in language use when lying is decreased first-person pronoun use [16–19], which appears to be compensated by increased third-person pronoun use [2,20]. This change in behaviour is argued to reflect liars' efforts to dissociate self from the lie and eschew personal responsibility for the event [2]. However, such an explanation is individualistic in origin in the sense that it assumes a liar will value the personal goal of avoiding self-incrimination at the expense of others. As Pennebaker [21] notes, the underlying mechanism that connects use of first-person pronoun with honesty is likely self-attention of the kind observed by Wicklund [22] in his studies of US undergraduate behaviour.

By contrast, collectivist cultures encourage interdependent selves, with interpersonal relationships and group well-being taking precedence over personal goals [13,23]. This impacts how speakers situate the person in their dialogue. For example, in their study of family dialogues, Ng *et al*. [24] found that the use of single versus multi-addressee turns at talk (i.e. those typified by single versus plural pronouns) correlated with the individualism versus collectivism of the family, and the acculturation of members within the family. Similarly, compared to those from individualistic cultures, those from collectivist cultures are more likely to drop personal pronouns from sentences in favour of highlighting joint perspectives and actions [25]. Thus, it is not clear that the reduction in first-person pronoun use shown by Western student populations when lying will be present across all cultures. It seems plausible that individuals with collectivist backgrounds will be more concerned with reducing the extent to which others are associated with the lie, by focusing the account they provide away from others and onto self. This possibility implies a differential use of references to self and other across cultures: liars from individualistic cultures will use fewer first-person and more third-person pronouns to distance self from the deceit, while liars from collectivist cultures will use more first-person and fewer third-person pronouns to distance the social group from the deceit (hypothesis 1).

A second key finding from the deception literature is that fabrications lack the contextual details that occur naturally when individuals recall from memory [3]. Contextual details are the sensory and relational descriptors that enrich an account by associating events with objects, actors and locations [3,26]. However, the way in which individuals sample, process and recall information from the environment has long been known to vary across individualism–collectivism [23,27,28]. Consistent with existing results in the deception literature, the reported memories of those from individualistic backgrounds are heavily tied into individual-based experience such that stories are told relative to objects and perceptual stimuli that are personally seen, felt and understood [29].

By contrast, a collectivist's reporting is tied to group actions and outcomes and so may be expected to emphasize social inter-connections and relationships among actors [30]. For example, Wang [31] demonstrated that priming Asian Americans with their individualistic American-self or collectivist Asian-self determined whether they reported more self-focused details, such as references to personal emotional and judgements, or more socially orientated memories, such as references to relationships and group actions. Paspalanova [32] has shown that this difference in recall holds true for episodic memories of a controversial social event that is high in emotive content. Those greater in collectivism tended to report aspects of the event in terms of relationships rather than sensory details, while it is sensory details that are prominent in the memories of the more individualistic participants. Taken together, this evidence suggests that individuals from collectivist cultures may tend to use a more relational or socially oriented form of contextual embedding in their genuine accounts. Thus, while lies by those from individualistic cultures would be predicted to include fewer instances of reported perceptual information compared to truths (as found in previous research), lies by those from collectivist cultures would be predicted to include fewer instances of social embedding compared to truths (hypothesis 2).

A third area in which research has identified predictors of deception is in relation to affective cues. Liars have been shown to present more negative affect than truth-tellers, which is typically attributed to liars feeling guilty and anxious about their deceit [3,33]. However, yet again, the expression of emotion has been shown to vary across culture, with several converging lines of evidence suggesting that liars from collectivist cultures will not show affect in the way demonstrated in existing studies with individualistic cultures. For example, Matsumoto *et al*. [34] found a strong positive correlation between individualism and emotional expression, indicating that restraint in emotional expression is a more normative and practised response for collectivists. Moreover, compared to individualistic cultures, the experience within collectivist cultures is one of more socially engaged emotions (e.g. friendly) than socially disengaged emotions (e.g. anger) [23]. This is due, in part, to the fact that positive affect is utilized in collectivist cultures to avoid conflict and ensure in-group harmony [35]. Those from collectivist cultures report lying as a more socially acceptable behaviour [36], suggesting that it may not provoke the guilt that is associated with negative affect among individualistic liars. Thus, this evidence suggests that individuals from collectivist cultures will be less likely to show negative affect when they lie, and that they may, in fact, present with positive affect in order to maintain social harmony (hypothesis 3).

### 1.2. Current study

The current study tests the hypotheses described above in statements given by participants from four ethnic groups. Specifically, we examine the relative use of seven linguistic categories that map directly onto the aspects of language use that underlie the predictions. Our focus on seven categories is deliberate and reflects the fact that we are seeking to test specific hypotheses rather than explore the best predictors of lying in different cultural groups. While it is possible to compare cultural groups across a larger set of linguistic predictors, doing so is likely to be susceptible to Type I error, particularly given the complexities of cultural norms. Thus, using as few variables as possible to minimize the occurrence of false positives, our approach was to test hypotheses that both stand out in the cross-cultural literature as likely areas for differences and that are central to methods of lie detection popular in current practice (e.g. criteria-based content analysis [8,37]).

## 2. Material and methods

### 2.1. Participants

Participants were recruited from community and religious centres across North West England. We identified contact details for 364 centres through local government information and Internet searches. These centres were approached by post (*n* = 249), by telephone (*n* = 75) or by email (*n* = 40) and invited to participate in the research. Of these, 56 responded to the initial invitation, either declining to take part or expressing interest (for many, the contact information was no longer correct (e.g. because they had closed)). Those who declined to take part (*n* = 37) typically did so because they felt that their members would not be interested in taking part, because the logistics of organizing a session was not possible, or because they primarily worked with children rather than adults. Those who responded positively (*n* = 19) were met by a member of the research team who explained the purpose and logistics of the research and who agreed a date(s) for data collection.

The final data comprised responses from 345 participants who were recruited from 19 centres (nine representing Black Africans, two representing South Asian, six representing Eastern European and two representing White British). Of these, 25 were removed from the final data because they failed to follow the instructions, either by not lying when required (*n* = 8) or by providing incomplete answers (mean words = 28.3, range 15–41). Of the remaining 320 participants, 174 were women. When asked their age bracket, 105 reported 16–25 years, 91 reported 25–34 years, 47 reported 35–44 years, 35 reported 45–54 years, 15 reported 55–64 years and 27 reported above 65 years.

Participants self-identified their ethnic background as being either Black African (*n* = 80, 41 women), South Asian (*n* = 80, 29 women), White European (*n* = 80, 52 women) or White British (*n* = 80, 52 women). With the exception of the White British group, participants also reported being foreign-born, first-generation immigrants to the UK. Those indicating Black African ethnicity reported being from Ethiopia (2), Ghana (9), Mozambique (7), Nigeria (9), State of Eritrea (2), the Republic of Congo (9) and Zimbabwe (42); those indicating South Asian ethnicity reported being from Bangladesh (32), India (3) and Pakistan (45); those indicating White European ethnicity reported being from Bulgaria (2), Hungary (1), Lithuania (14), Poland (61), Romania (1) and Slovakia (1); and those indicating White British ethnicity reported being from England (80). There were no significant differences across the cultural groups in terms of sex, $\chi_{\left(3\right)}^{2}=7.74$, *p* = 0.053, *V* = 0.16, 95% CI [0.07, 0.38], but there were differences in average age. The mean rank of age bracket reported by South Asian participants was lower (i.e. younger) than the other three groups, *U*(1) = 4743.0, *z* = −7.00, *p* < 0.001, *θ* = 0.25, 95% CI [0.19, 0.31], while the mean rank of age bracket reported by White British participants was higher (i.e. older) than the three other groups, *U*(1) = 3219.0, *z* = −9.20, *p* < 0.001, *θ* = 0.17, 95% CI [0.13, 0.23]. These differences do not relate systematically to the findings of the hypothesis tests reported below and so they are unlikely to account for these effects.

Although we acknowledge that there will be ethnic variation across and, indeed, within country groups, we retain participants' self-identified groupings for two reasons. First, the groups reflect categories that are employed in many national censuses (e.g. the US Census, UK national census) and so they are categories that impact government and law enforcement policy decisions [38]. Second, the groupings have empirical value. Specifically, the four groupings span the different world regions reported in Oyserman *et al*.'s [29] meta-analysis of individualism and collectivism. Oyserman *et al*.'s effect sizes for the deviation away from European American individualism orders the groups from White British (‘English-speaking’, *d *= 0.05) to White European (‘Central Europe’, *d* = 0.12) to South Asian (‘Other Asia’, *d* = 0.18) to Black African (‘Africa’, *d* = 0.39). Collectivism, examined independently by Oyserman *et al*., orders the groups in reverse, from Black African (*d* = −0.77), to South Asian (*d* = −0.41) to White European (*d* = −0.22) to White British (*d *= −0.06).

### 2.2. Materials and procedure

On giving informed consent, participants of the same ethnicity completed the study in pairs within a quiet area of their community centre. The pairs were formed at random and not matched on any demographic characteristic (e.g. sex or age). Participants were informed that the first part of their task was to individually complete an experimental booklet. The booklet informed participants that their task was to provide a genuine and fabricated account, and that they should try to make their accounts appear genuine because their partner would be given the accounts and asked to identify the fabrication. They were told that they as an individual would receive £5 and that their local venue would also receive £5 if they duped their partner into believing that their fabrication was genuine. This payment was intended to raise the personal stakes (potentially more motivating for individualist participants) and social stakes (potentially more motivating for collectivist participants) associated with producing convincing accounts [39,40]. In reality, all participants were paid £10.

The accounts that participants were asked to provide differed according to their random assignment to one of two conditions. These conditions were based on tasks that have proven successful in past research [2,17,33,41,42]. In the ‘Experience’ condition, participants were asked to describe an event that occurred within the last year, and a fabricated experience that did not occur (they were asked to ensure their account was entirely fabricated and not, for example, based on truth with some details changed). In the ‘Opinion’ condition, participants were asked to write in support of a topic about which they felt strongly and then to provide an equally convincing account for the opposing viewpoint. In both conditions, participants were given examples of possible topics; for example, in the opinions condition they were given the examples ‘smoking in public places’, ‘same-sex marriage’ and ‘the death penalty’.

Our use of two lie conditions stems from a recognition that different kinds of lie may elicit different language use [26,43]. For example, it may be that lies about experience lack the spatial and temporal details that typify a true account, while lies about opinions lack the emotive content associated with a passionate belief in a position. Comparing language use across different kinds of lie makes it possible to consider the extent to which differences in liar behaviour may generalize across contexts.

Participants wrote their accounts in the booklet individually, in their own time, and in English. An experimenter remained in the room during this period to ensure pairs did not discuss the task or their response. The order in which they were asked to provide the two accounts was counterbalanced (no effects were found). At the end of the booklet, they were asked to provide demographic information. Once both members of a pair had completed their booklet, which took approximately 15 min, they exchanged booklets and were encouraged to make judgements about the veracity of their partners' accounts. After rendering their judgement, the pairs were given the opportunity to reveal the correct answers, and they were debriefed.

### 2.3. Analysis of linguistic features

To derive measures of language use, typed versions of participants' responses were analysed using the text analysis program Linguistic Inquiry and Word Count (LIWC [44]). For each of the participants' statements, we used LIWC to calculate the percentage of words that related to six categories relevant to our hypotheses: first-person and third-person pronoun (e.g. *I*, *they*), positive and negative affect words (e.g. *amazing*, *grief*) and perceptual and social details (e.g. *sharp*, *interact*). (For perceptual we combined the LIWC categories *Perceptual* and *Relativity* to provide a more complete measure of descriptor of objects and their relationships.) The focus on percentage of occurring category words (i.e. category occurrence/word count), rather than on an absolute count of occurring words, ensures that the category scores are not a direct function of statement length. These LIWC categories, which are described in detail by Pennebaker *et al*. [45], have been widely used in applied psychology [17,46,47] and shown to provide reliable and valid analysis of written and spoken texts [48,49]. An account of LIWC's reliability and further examples of the words contained within each category are given in Pennebaker *et al*. [45].

## 3. Results

### 3.1. Preliminary checks

We conducted a preliminary analysis to determine whether or not participants' language proficiency might account for effects observed across the ethnic groups. Because of time constraints, it was not possible to conduct a formal evaluation of language proficiency. Instead, we drew on two measures that could be applied to the participants' responses directly. First, we examined the Flesch–Kincaid grade level score, which uses sentence length and syllables per word to estimate the US educational level at which individuals would typically understand 75% of the text [50]. As might be expected, Flesch–Kincaid scores are associated positively with writing proficiency [51]. A one-way ANOVA contrast revealed no significant decrease in Flesch–Kincaid scores across the individualism–collectivism groupings for the genuine statements, *t*_316_ = −1.28, *p* = 0.202, nor for the fabricated statements, *t*_316_ < 1, *ns*.

Second, we examined aspects of the language quality of the texts using Coh-Metrix [52,53]. Coh-Metrix produces a range of indices that capture text coherence, defined as the extent to which the language of the text is sufficient to create a coherent mental representation in the listener. We examined four measures that capture variation at different layers of language representation and processing [54], namely: (i) lexical diversity (LDTTRc) to capture differences in the range of words used; (ii) syntactic simplicity (PCSYNp) to capture the extent to which simple, familiar syntactic structures are used; (iii) semantic cohesion (LSASS1) to capture the degree of conceptual similarity expressed over the text; and (iv) situational model references (SMCAUSv) to capture differences in the degree the texts provided a listener with a causal understanding of the event. A set of ANOVA contrasts across the individualism–collectivism groupings suggested there was no systematic change across the four cultural groups in: lexical diversity for the genuine, *t*_316_ < 1, *ns*, and fabricated statements, *t*_316_ < 1, *ns*; syntactic simplicity for the genuine, *t*_316_ < 1, *ns*, and fabricated statements, *t*_316_ = 1.20, *p* = 0.23; semantic cohesion for the genuine, *t*_316_ < 1, *ns*, and fabricated statements, *t*_316_ = −1.37, *p* = 0.17; and situational model for the genuine, *t*_316_ < 1, *ns* and fabricated statements, *t*_316_ < 1, *ns*. The absence of differences across these measures suggests that there were no variations in the way in which each of the four cultural groups used language that could explain the differences observed at hypothesis testing.

Finally, although LIWC takes account of statement length by using percentage scores, for completeness we computed a 4 (culture) × 2 (veracity) × 2 (type of lie) mixed ANOVA in which statement veracity was a within-participant factor and word count was the dependent variable. This revealed a main effect of veracity, with participants' truthful accounts containing on average more words (*M* = 156.95, s.d. = 80.99) than participants' fabrications (*M* = 139.37, s.d. = 75.86), *F*_1,312_ = 34.05, *p* < 0.001, *η*^2^ = 0.10, 95% CI [0.04, 0.16]. However, the difference between genuine and fabricated statement length did not vary across the cultural group, *F*_3,312_ = 1.40, *p* = 0.243, *η*^2^ = 0.01, 95% CI [0.00, 0.04], or type of lie, *F*_3,312_ = 2.34, *p* = 0.127, *η*^2^ = 0.01, 95% CI [0.00, 0.06], suggesting that word count would not explain the differences observed across cultural groups in our hypothesis testing.

### 3.2. Hypothesis testing

To focus the hypothesis testing on change in behaviour across genuine and fabricated statements, we computed difference scores. Specifically, for each participant's scores on each category, we subtracted the proportion found in their genuine account from the proportion found in their fabricated account. A positive score therefore indicated greater use when deceiving than when telling the truth. These difference scores were submitted to a series of 4 (culture) × 2 (type of lie) × 2 (category) mixed ANOVA, in which category was a within-participant factor that related to the language categories being compared. A table of original means and standard deviations for the linguistic categories as a function of ethnic group is given in electronic supplementary material 1, descriptive statistics for linguistic cues.

**Figure 1.**
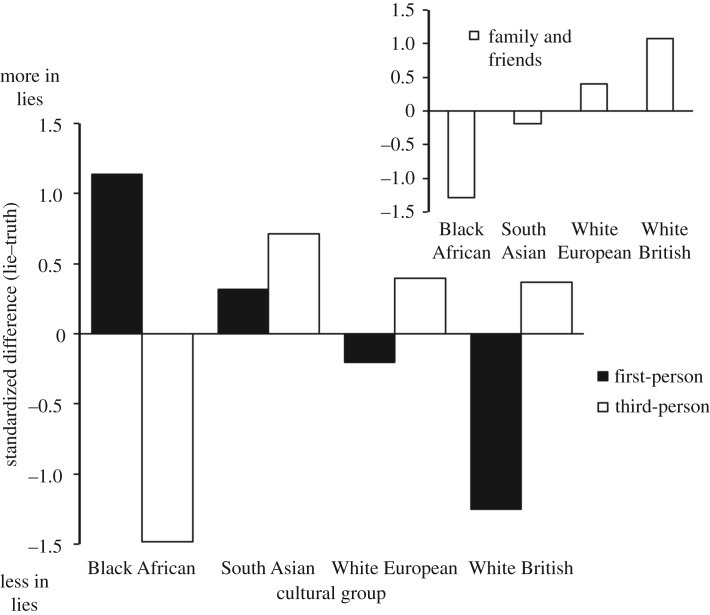
Standardized difference scores (%-in-fabricated minus %-in-truthful) for first- and third-person pronoun use as a function of the ethnic group. The difference scores are standardized to enable a comparison across pronoun type. Figure inset: standardized difference scores for the sum of the family and friend category.

We predicted an opposite pattern of first- and third-person pronoun use across participants from individualist and collectivist cultures (hypothesis 1). A culture × lie type × pronoun type interaction, *F*_3,312_ = 3.92, *p* = 0.009, *η*^2^ = 0.04, 95% CI [0.003, 0.078], confirmed significant cultural differences in the way participants changed their pronoun use when lying compared to when telling the truth. As shown in figure 1, compared to their use in truthful statements, first-person pronouns were used the most by North African participants when lying and the least by White British participants when lying, while the opposite pattern was true for third-person pronoun use. A planned pronoun type × culture interaction contrast, *F*_1, 316_ = 3.04, *p* = 0.041 one-tailed, *η*^2^ = 0.01, 95% CI [0.000, 0.041], provided evidence that increased/decreased use of first-person pronouns when lying was associated with decreased/increased use of third-person pronouns when lying. A deconstruction of this interaction contrast across lie type, prompted by the initial three-way interaction, revealed that this interaction trend existed for lies about experience (*r*_first-person_ = −0.15, *r*_third-person_ = 0.16), *F*_1,156_ = 7.64, *p* = 0.006, *η*^2^ = 0.05, 95% CI [0.004, 0.125], but not for lies about opinions (*r*_first-person_ = 0.01, *r*_third-person_ = −0.01), *F *< 1.

As shown in figure 1, the trend in third-person pronoun use across groups is not as clear as that for first-person pronoun use. One explanation for this pattern of results is that third-person pronoun use can refer to a range of people, from those within a participant's immediate social circle to those socially remote. It may be that the predicted effect will be more pronounced when participants are referring to their immediate social circle (e.g. *husband*, *girlfriend*). To explore this possibility, we examined the change in reference to family and friends using a composite variable of LIWC's Family and Friend categories (see inset in figure 1). Consistent with the predicted differences in third-person pronoun use, this refined variable was associated with a significant linear trend through Black African, South Asian, White European and White British groups, *F*_1,316_ = 5.07, *p* = 0.026, *η*^2^ = 0.03, 95% CI [0.000, 0.053].

**Figure 2.**
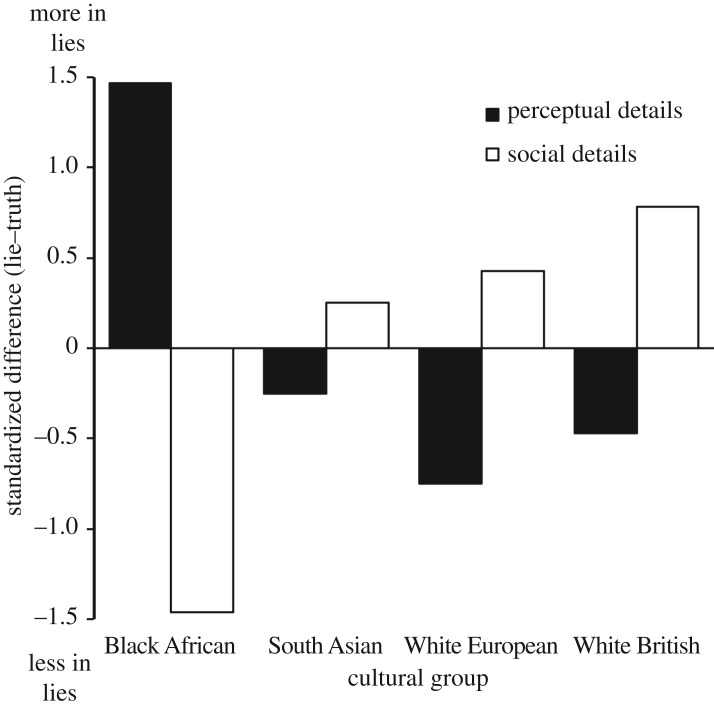
Standardized difference scores (%-in-fabricated minus %-in-truthful) for perceptual and social details as a function of ethnic group.

Our second prediction concerned the nature of contextual embedding across cultures (hypothesis 2). The mixed ANOVA revealed a significant culture × lie type × embedding type interaction, *F*_3,312_ = 2.85, *p* = 0.037, *η*^2^ = 0.03, 95% CI [0.000, 0.063], confirming the existence of cultural differences in the way in which context was described during genuine and fabricated statements. As shown figure 2, when comparing across the four groups (from Black African through to White British), there is a trend of providing fewer perceptual details and more social details when lying. A significant planned contextual embedding × culture interaction contrast, *F*_1, 316_ = 7.30, *p* = 0.007, *η*^2^ = 0.02, 95% CI [0.002, 0.064], confirmed that increased/decreased use of perceptual-relative words when lying was associated with decreased/increased use of social words when lying. A deconstruction of this interaction across lie type, prompted by the initial three-way interaction, revealed that this interaction trend existed for lies about experience (*r*_perceptual_ = 0.15, *r*_social_ = −0.25), *F*_1,156_ = 11.51, *p* = 0.001, *η*^2^ = 0.07, 95% CI [0.012, 0.155], but not for lies about opinions (*r*_perceptual_ = 0.003, *r*_social_ = 0.06), *F *< 1.

Our third hypothesis predicted cultural differences in the use of positive and negative affect when lying compared to when telling the truth (hypothesis 3). The culture × lie type × affect type ANOVA revealed a main effect of affect type, *F*_1,312_ = 10.19, *p* = 0.002, *η*^2^ = 0.03, 95% CI [0.005, 0.078], with participants regardless of culture using more positive affect words when lying compared to when telling the truth (*M*_difference_ = 0.50) and fewer negative affect words when lying compared to when telling the truth (*M*_difference _= −0.27). This main effect was qualified by an affect type × lie type interaction, *F*_1,316_ = 4.94, *p* = 0.027, *η*^2^ = 0.02, 95% CI [0.000, 0.052], which revealed that the differences in affect word use was true for lies about opinions (*M* diff._Positive affect_ = 0.75, *M* diff._Negative affect_ = −0.57), *t*_159_ = −3.29, *p* = 0.001, *d* = −0.52, 95% CI [−0.838–0.203], but not lies about experience (*M* diff._Positive affect_ = 0.26, *M* diff._Negative affect_ = 0.02), *t* < 1.

## 4. Discussion

The results demonstrate that linguistic cues to deception do not appear consistently across cultures. In relation to pronoun use, we found evidence for a shift towards using fewer first-person pronouns, and more third-person pronouns, when moving across Black African, South Asian, White European and White British groups. In relation to contextual embedding, we found evidence of a shift towards including fewer perceptual details, and more social details, when moving across Black African, South Asian, White European and White British groups. Critically, these changes in behaviour are consistent with previously identified differences in how individuals from individualist and collectivist cultures construe self [23] and report memories of events [27]. Thus, as found in other areas of psychology [5,6], our findings suggest that the use of Western participant groups has led to an over-simplification of thinking about how people behave when they lie. Our data suggest that culture affects who liars seek to protect through their deceit and the kinds of contextual details that are absent in their fabrications.

In contrast to these differential trends, we found that neither the degree nor valence of affect-related language use differed significantly across cultures. This finding is interesting when compared with evidence of a strong correlation between individualism and expressiveness [34]. It implies, given the absence of a culture main effect, that use of emotive language during deception may have strategic rather than ‘leakage’ roots. Compared to those whose cultural norm is for direct displays of emotion, we might expect those whose norm is for modest and restrained displays of emotion to show less pronounced affective behaviour [23]. This may be particularly true when lying, because lying can be stressful and research suggests that stress exacerbates normative behaviour [55,56]. The absence of such a difference in our data suggests that participants were choosing to present positive affect, perhaps to maintain social harmony [36]. While this explanation is speculative—it is an interpretation of a null effect—it is interesting that this shift in affect has been found elsewhere in scenarios where liars have sufficient time to use emotion strategically [19]. More generally, as Hatz and Bourgeois [57] show in their analysis of anger in lies, our findings demonstrate the need to be specific about the exact kind of emotion under examination.

A third finding to emerge from our study relates to the moderating effect of lie type. We found that pronoun use and contextual embedding varied when participants lied about experiences but not when they lied about opinions. By contrast, participants' affect-related language varied when they lied about opinions but not experiences. These effects of context are consistent with trends in the literature. For example, studies demonstrating the diagnostic value of contextual embedding have tended to compare truths and lies about observed or experienced events [3,40]. By contrast, studies demonstrating the value of emotion as a cue have tended to compare truths and lies about opinions ([2,10]; cf. [33]). Thus, the relevance of different explanations for why cues to deception occur, namely those focusing on cognition-related factors (e.g. memory) versus those focusing on anxiety-related factors (e.g. emotion), may differ according to the type of lie required by the context. This explanation complements recent efforts to identify interview questions that make lying difficult [26], since it suggests that different kinds of questions may be most appropriate for different kinds of lie.

Collectively, our findings emphasize the importance of developing a richer understanding of what it means to lie. The variations found across our cultural groups appear to be grounded in different social motivations for how memories are encoded and for how they are presented when lying. This suggests that it may not be sufficient to conceptualize a liar as somebody motivated ‘to not get caught’, since liars may pursue different social goals when deceiving, which influences both what they seek to conceal or fabricate, and how they go about doing so [36]. Equally, how liars behave also appears to vary by task, as illustrated by our finding that the cues useful for detecting a liar differed depending on whether the lie was about an experience or an opinion. Thus, there are both motivational and experiential differences to ‘not getting caught’. Understanding these in more detail should afford a better formulation of current theoretical explanations (e.g. cognitive load [26]) for why people ‘leak’ deceptive behaviour.

The possibility that deceit manifests differently across cultures is important to law enforcement policy in contexts such as police interviews [4], airport screenings [43] and hostage negotiations [58]. For example, SCAN, a method popular with federal law enforcement and military agencies [1], encourages analysts to examine a statement for the use of first-person, past-tense pronouns, which associates positively with truth. Our findings suggest that analysts would need to interpret this criterion in different and nuanced ways depending on the cultural background of the suspect. For example, in contrast to the current SCAN guidelines, greater use of first-person pronouns by a North African suspect should be weighted as indicative of lying. Such complexities make it difficult to give definitive recommendations about the kinds of behavioural patterns, and associated SCAN scores (i.e. cut-off values), that would indicate that a statement is deceptive beyond reasonable doubt.

A similar argument may be made about language analysis in court proceedings. Statement validity analysis has been accepted as evidence in relation to a child's credibility in some US courts [59] and in criminal courts in Europe [60,61]. One component of statement validity analysis, CBCA, uses criteria for differentiating truth from lies that include the degree of contextual embedding given in the statement, and categories that focus on perceptual details (e.g. unusual details) and events as they happened to the ‘remember’ (e.g. descriptions of interactions). Arguably, these three criteria align with the language features examined in our study [19]. Thus, our evidence, if found to replicate in a child sample, suggests that the definitions of these criteria would need adapting to encompass relational memories.

It is also important to other legal scenarios that seek to establish or rely on authenticity, such as forensic risk assessments [62], discrimination proceedings [63] and the evaluation of asylum seekers [64]. In the absence of culture-specific training, an individual's judgements about veracity is most likely drawn from either experience or an evidenced-based understanding based on studies of Western liars. In these scenarios, erroneous judgements of veracity may impact on justice. For example, in Bond and Atoum's [65] demonstration of poor deception judgement accuracy across cultures, the condition that showed the worst decline in performance was participants who heard the suspect's verbal behaviour but could not see their non-verbal behaviour. Our findings point to one of the mechanisms that may lead to this difference in perceptions, namely, a different linguistic form of presentation. This is consistent with Da Silva and Leach's [66] demonstration that second language users are judged through the lens of a ‘lie bias’ rather than a ‘truth bias’, as typically afforded native users of English. When culturally normative behaviour contradicts an individual's expectations about honest behaviour, it may lead to misplaced suspicion, which may have a negative impact on relationships and perceptions of fairness of the assessment process [67,68].

While our focus on broad ethnic groups provides an important examination of deception across cultures, it ignores the differences that exist between and within countries and communities. Our findings are therefore likely to be an overgeneralization of actual differences in behaviour, and future research may reveal variations and other individual differences that modify and even contradict the simple contrasts found here [29,69]. As the specificity of the samples increase, so we should expect to be able to find stronger effects (i.e. because the error of combining variance over cultures is removed) and be able to focus on a broader range of linguistic variables (i.e. because some will be relevant to particular cultures but not others). We should also anticipate these effects being moderated by other factors, such as the country in which the research is conducted. Participants living among their home culture may show stronger differences than participants whose ‘heritage’ culture has since been influenced by the culture into which they have immigrated. The fact that we found significant patterns in behaviour despite the focus on broad ethnic groupings and only a few linguistic variables suggest that there are important effects to discover in this area.

It is important to acknowledge that the effects found for the cross-cultural trends in behaviour are small and would likely be difficult for an interviewer to perceive. Several aspects of the research design may account for these small effects. For example, although we gave participants instructions to provide complete lies, it was not possible for us to check that they complied with this instruction. This leaves open the possibility that some lies were ‘couched’ within broader truths, thereby limiting the changes in language that could be observed. Similarly, participants were given free-reign over what they reported. It is possible that participants provided statements that were qualitatively different, perhaps because of their cultural background or to minimize the difference between their truth and lie. For example, participants high on collectivism may have given preference to recounting family events, while participants high on individualism may have given preference to their personal achievements. We limited the impact of such differences by comparing the genuine and fabricated statements of the same participant; there is no reason to expect participants to show a preference in only one of their two stories. Nevertheless, there may be subtle qualitative differences at play even within multiple statements from a single participant, and the impact of such differences on language use requires attention.

The current results may also be limited by the fact that our methodology ‘fixed’ the way in which participants lied. As Triandis *et al*. [70] observe, silence can provide a useful way to deceive within collectivist groups, and our participants were not afforded this option in their statements. Similarly, our methodology is lacking some of the ‘contextual’ factors, such as interviewer cues and life-changing stakes, which may further shape the way in which a suspect's culture affects their behaviour [5]. The absence of an interviewer is particularly salient to any interpretation of our findings, given Wang's [31] demonstration that priming alternative self-views can alter the nature of autobiographical memory. For example, Wang's findings imply that a typical UK police interview context and a White British police interviewer would prime an autonomous self-concept within a South Asian immigrant. This would lead them to report more self-focused memories than would otherwise be the case, thus diminishing the differences in linguistic behaviour observed in this study.

These limitations notwithstanding, our findings identify one reason why researchers struggle to find a consistent set of linguistic indicators of deception [3]: their findings may have been influenced by the cultural heterogeneity of the participant group. In today's world, where law enforcement and justice are asked to respond to a greater cultural diversity of suspect [58], it will be important to use findings such as those presented here to adapt existing practices and policies so that they afford justice for all communities within the population.

## Supplementary Material

Descriptive Statistics

## Data accessibility

Data and scripts supporting this article are deposited at Dryad: (http://dx.doi.org/10.5061/dryad.45jq5) [71].
