# Correction of ‘Culture moderates changes in linguistic selfpresentation and detail provision when deceiving others’

**DOI:** 10.1098/rsos.201803

**Published:** 2020-10-28

**Authors:** Paul J. Taylor, Samuel Larner, Stacey M. Conchie, Tarek Menacere

*R. Soc. open. sci*. **4**, 170128. (Published 7 June 2017). (doi:10.1098/rsos.170128)

In 2019, the journal was made aware of a concern regarding the clarity of the funding and competing interest statements supplied with a published paper during the review process. The readers contacted the journal to indicate they considered that a potential conflict of interest may exist that had not been adequately disclosed.

The readers considered a potential conflict of interest to exist in relation to the proportion of the funding for this research derived from an ESRC grant (ES/N009614/1, https://gtr.ukri.org/projects?ref=ES%2FN009614%2F1). This grant was awarded to the Centre for Research and Evidence on Security Threats (CREST), which is a national hub for understanding, countering and mitigating security threats. It is an independent centre. In the original competing interest and funding statements supplied, it was not made clear that CREST (through the grant above) is funded in part by the UK security and intelligence agencies.

While the decision to issue a correction was made by the editorial team, advice was sought from the Committee on Publication Ethics (COPE). With this support, a revised statement has been written to provide greater clarity, and so the funding statement is amended to ‘A proportion of the funding for this research derived from an ESRC grant (ES/N009614/1, https://gtr.ukri.org/projects?ref=ES%2FN009614%2F1), which was partly supported by the UK security and intelligence agencies. This grant was awarded to the Centre for Research and Evidence on Security Threats (CREST), which is a national hub for understanding, countering and mitigating security threats. It is an independent centre.’

Similarly, the competing interest statement is amended to reflect the fact that outputs from CREST are reviewed prior to submission for publication by a designated contact for the intelligence and security services. However, the authors note submission to the designated contact is a service to authors to ensure they do not break the law by willingly or unwillingly disclosing sensitive or classified information that could have a detrimental impact on national security, and that no changes were requested prior to or during submission of the paper to Royal Society Open Science. The competing interest statement is amended to ‘Under the grant conditions, the UK intelligence and security services have the right to examine and request the removal of sensitive information from research to be submitted for publication under this grant. The authors declare that, while the paper was supplied to a designated contact for review by the UK intelligence and security services before submission to Royal Society Open Science, no redaction or amendments were asked for or made.’

This correction was prepared by the journal's editorial office, with the text approved by the original paper's authors.

