# Supplementary material for: Culture moderates changes in linguistic self-presentation and detail provision when deceiving others
Source: R Soc Open Sci. 2017 Jun 7;4(6):170128. doi: 10.1098/rsos.170128 (PMC5493910; doi:10.1098/rsos.170128)
Supplement: Descriptive Statistics [file rsos170128supp1.docx]

Supplementary Material 1.

*Table Containing the Means and Standard Deviations (in parentheses) for the Linguistic Categories as a Function of Ethnic Group*

|  | Ethnic Group | | | | | | | |
| --- | --- | --- | --- | --- | --- | --- | --- | --- |
| Linguistic Category | Black African | | South Asian | | Eastern European | | White British | |
|  | T | F | T | F | T | F | T | F |
| First Person Pronoun | 5.44  (4.47) | 6.58  (5.64) | 5.08  (4.81) | 5.94  (5.57) | 5.58  (4.17) | 6.22  (5.18) | 2.69  (2.49) | 3.08  (3.34) |
| Third Person Pronoun | 2.78  (2.41) | 2.31  (2.66) | 2.41  (2.63) | 2.73  (2.75) | 2.61  (2.58) | 2.81  (2.99) | 1.76  (1.90) | 1.95  (1.85) |
| Family and Friends | 1.40  (1.79) | .82  (1.41) | 1.15  (1.45) | .82  (1.43) | 1.08  (1.40) | .96  (1.23) | .78  (1.19) | .70  (1.06) |
| Perceptual details | 15.70  (5.79) | 16.62  (7.52) | 16.65  (7.67) | 15.72  (6.52) | 16.61  (7.44) | 15.98  (6.77) | 16.43  (5.90) | 16.32  (5.85) |
| Social details | 12.94  (5.60) | 10.40  (5.53) | 11.65  (5.30) | 11.42  (5.46) | 10.36  (5.32) | 10.48  (6.09) | 9.54  (3.88) | 9.75  (4.63) |
| Positive Emotion | 3.24  (2.56) | 3.35  (2.43) | 3.10  (2.89) | 3.14  (2.74) | 3.62  (2.30) | 4.77  (3.81) | 2.84  (1.71) | 3.41  (1.98) |
| Negative Emotion | 1.82  (2.04) | 1.65  (1.83) | 2.41  (3.05) | 2.41  (2.17) | 2.48  (2.18) | 1.79  (1.74) | 1.78  (1.75) | 1.62  (1.46) |

Note: T = Truth; F = Fabrication.
